# Photoluminescence Mechanism of Carbon Dots: Triggering Multiple Color Emissions through Controlling the Degree of Protonation

**DOI:** 10.3390/molecules27196517

**Published:** 2022-10-02

**Authors:** Hao Yi, Jing Liu, Jian Yao, Ruixing Wang, Wenying Shi, Chao Lu

**Affiliations:** State Key Laboratory of Chemical Resource Engineering, Beijing University of Chemical Technology, 15 Beisanhuan East Road, P.O. Box 98, Beijing 100029, China

**Keywords:** m–phenylenediamine, protonation, carbon dots, photoluminescence mechanism, tunable photoluminescence

## Abstract

Carbon dots (CDs) have excellent optical properties, low toxicity and easy preparation, which have led to them being widely used in biomedicine, sensing and optical devices. However, although great progress has been made in the preparation of CDs, the detailed exploration of their photoluminescence (PL) mechanism is still under debate due to their complex structures and surface functionalities. Here, we proposed a single change in the pH of the synthesis condition, which had no effect on the CDs intrinsic core states and avoided the mutual influence of multiple PL origins. The m-phenylenediamine (m–PD) served as a carbon source, whose protonation degree determined the surface state of the resulting CDs and the accompanying fluorescence characteristics. The as-obtained CDs materials can be applied in the chemical sensor and anti-counterfeiting fields in a targeted manner. Therefore, our work not only contributes to the explanation of the CDs PL mechanism, but also obtains a series of CDs materials with controllable PL properties.

## 1. Introduction

Quantum dots are unique nanomaterials whose intrinsic properties are altered by quantum effects. They have broad application prospects in photovoltaic devices, biomedicine and sensing [1,2]. Among them, carbon dots (CDs) have aroused extensive research interest since their discovery in 2004 [3]. New, impressive progress of CDs is made every day, which benefits from their many advantages: (1) CDs possess tunable photoluminescence (PL) and high environmental resistance (photo and heat) [4]. More attractively, the PL quantum yields (PLQYs) can even reach 100% [5,6,7]; (2) from the point of view of raw materials and preparation methods, it is easier to obtain CDs at low costs [8]. Various carbon-containing raw materials are used as precursors to achieve CDs by various preparation methods [9]. Raw materials include amino acids, citric acid, glucose, graphite and its derivatives, plants, coal, hair, grass, fruit, petroleum coke and even domestic waste [10,11,12]. Preparation methods include chemical oxidation, hydrothermal, pyrolysis, microwave and so on [13,14]; (3) the abundant surface groups endow CDs with good dispersion and stability in water. More interestingly, their chemical polarity can be easily regulated by choosing suitable precursors, favoring dispersion in more solvents [15,16]. However, although great progress has been made in the preparation of CDs, the detailed exploration of their PL mechanism is still under debate due to their complex structures and surface functionalities [17]. Therefore, it is very urgent to have a better understanding for the PL origin, so as to usher in the progress in their applications relying on PL changes. 

In the past decade, due to the continuous efforts of researchers, great progress has been made in understanding the PL mechanism of CDs. Presently, the primary viewpoints can be summed up as the surface state, carbon core state, molecule state and their synergistic effect [18,19,20]. In 2006, Sun’s group firstly proposed the surface defect passivation mechanism based on the fact that the non-fluorescent carbon particles were converted into bright luminescence-emitting CDs through attaching simple organic species to their surfaces [21]. However, the surface state cannot explain the PL mechanism of some CDs with size-dependent PL or the same surface group but different fluorescence properties. Therefore, in 2013, Liu’s group came up with the intrinsic core states-related PL mechanism through the distinctly different emission from the sp^2^ and sp^3^ subdomains in comparison with the graphene quantum dots and graphene oxide quantum dots, respectively [22]. However, obviously, the model of the carbon core state is limited to only explain the CDs with a lattice structure, and really can not do anything about the CDs without a clear lattice structure [23]. Fortunately, in 2015, the pioneering work provided by Yang’s group proposed the specific molecular fluorophore-dominated PL center of CDs in citric acid as precursor [24]. However, from the perspective of the development of the PL mechanism of CDs, it is still in its infancy. Due to the fact that the raw materials and various methods of creating CDs determine the complexity of their compositions and structures, it is extremely difficult to compare with the results in the literature to formulate a unified PL mechanism [25,26]. Therefore, regulating the PL behavior of CDs under controlled and comparable conditions will be beneficial to determining the PL mechanism of CDs, and, consequently, obtaining the desired CDs luminescent material.

Due to the fact that the surface fluorophore of CDs usually contains acidic/basic functional groups, such as -COOH, -NH_2_, etc., the PL properties of CDs are very sensitive to external parameters, such as pH and the nature of the solvent medium. The change of pH value will change the degree of protonation on the surface of the CDs, which in turn affects their PL properties [27]. In order to explore the luminescence mechanism of the as-prepared CDs, we designed a relatively simplified experimental condition for the synthesis of CDs to minimize the interference factors. Here, we chose m-phenylenediamine (m−PD) as a model carbon source-molecule to fabricate the CDs by solvothermal method [28]. The amino group in m-PD is easy to be protonated and deprotonated via simply adjusting the pH value of the solution before the reaction [29]. The protonation degree of m-PD determines the surface state of the resulting CDs and the accompanying fluorescence emission characteristics. It is worth noting that by simply changing the pH value, only the degree of protonation of the functional groups (amino group) of the carbon source m-phenylenediamine (m−PD) molecule is changed, while the main skeletal structure of the molecule is not changed [30]. Therefore, this strategy not only contributes to the explanation of the PL mechanism of CDs, but also obtains a series of CDs materials with controllable PL properties, which can be applied in the chemical sensor and PL anti-counterfeiting fields in a targeted manner. 

## 2. Results and Discussion

### 2.1. Formation and Structure

**Figure 1:**
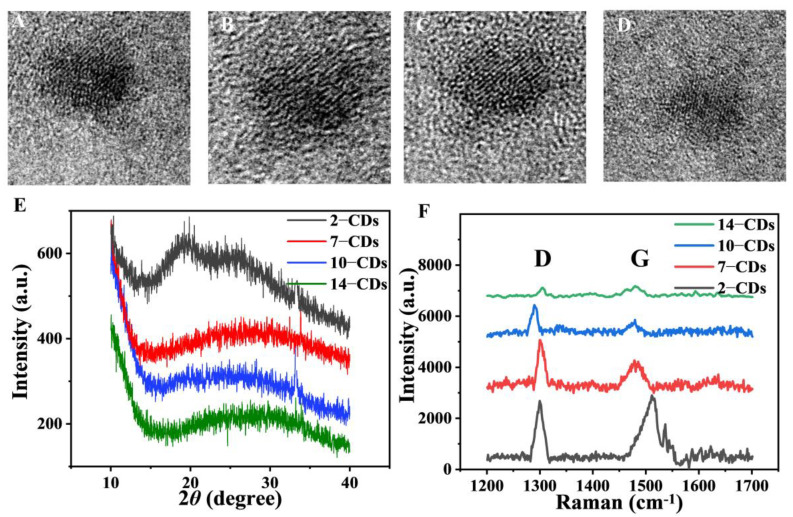
HRTEM images of the (**A**) 2–CDs, (**B**) 7–CDs, (**C**) 10–CDs and (**D**) 14–CDs. (**E**) XRD and (**F**) Raman spectra of the 2–CDs, 7–CDs, 10–CDs and 14–CDs (D peak representing C atom defect and G peak representing C atom stretching vibration).

To demonstrate the formation of CDs after a solvothermal treatment of m-PD with different degrees of protonation, high-resolution transmission electron microscopy (HRTEM), X-ray diffraction (XRD), Raman and transmission electron microscope (TEM) measurements were performed on the as-prepared products. The pH values of the synthesis conditions are 2, 7, 10 and 14, respectively, and the corresponding resulting CDs are named as 2–CDs, 7–CDs, 10–CDs and 14–CDs. Drops of the four CDs colloidal solutions were deposited on carbon-coated copper grids for HRTEM. The HRTEM images show that they have a spherical symmetry. The 2–CDs and 7–CDs have the lattice fringes of 0.22 nm, corresponding to the (100) facet of graphite (Figure 1A,B) [31], while the 10–CDs and 14–CDs have lattice fringes of 0.34 nm, corresponding to the (002) facet of graphite (Figure 1C,D) [32]. In the XRD patterns, 2–CDs, 7–CDs, 10–CDs and 14–CDs all have a broad amorphous peak at 20–30°, signifying the formation of a highly disordered C atom (Figure 1E). At the same time, in the XRD pattern of 2–CDs, a sharp and intense broad peak appears at 20°, indicating that the amorphous nature of C atoms is significantly increased [33]. Furthermore, in the Raman spectrum of Figure 1F, 1300 cm^−1^ represents the D peak of a sp^3^-hybridized C atom defect, and 1600 cm^−1^ represents the G peak of a sp^2^-hybridized C atom stretching vibration. The *I_D_*/*I_G_* ratios of 14–CDs, 10–CDs and 7–CDs are 0.986, 1.107 and 1.18, respectively. The above data show that with the deepening of the protonation degree, the more favorable it is for the generation of sp^3^ hybridized carbon atoms on the surface of CDs. However, 2–CDs does not conform to this law, where *I_D_*/*I_G_* ratios = 0.945. This is because the degree of conjugation (sp^2^) increases by a larger magnitude than the increase in surface states (sp^3^) [34]. Figure S1 presents the TEM images of the four CDs, along with particle size distribution experiments. The four CDs are relatively uniform spheres. From the distribution range, the highest proportions of 2–CDs, 7–CDs, 10–CDs and 14–CDs are 7.7 nm, 7.5 nm, 6.6 nm and 5.5 nm, respectively. Therefore, from a size perspective alone, the differences between the four CDs samples are not significant.

### 2.2. The PL Properties

**Figure 2:**
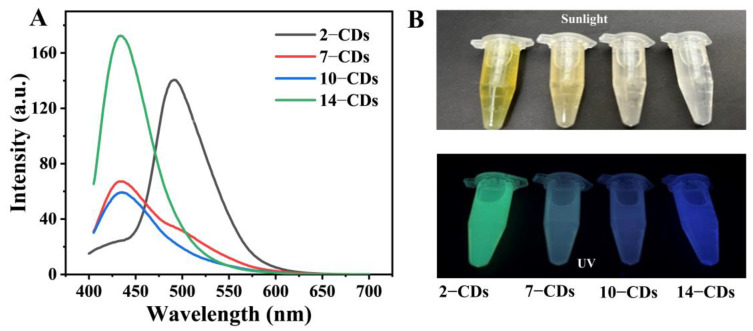
(**A**) Fluorescence emission spectra of the 2–CDs, 7–CDs, 10–CDs and 14–CDs. (**B**) Digital photo of the 2–CDs, 7–CDs, 10–CDs and 14–CDs in sunlight and under UV lamp.

In order to explore the influence of different protonation degree of a carbon source molecule m-PD on the PL properties of as-prepared CDs after solvothermal treatment, the steady-state spectra and quantum yields of the CDs (2–CDs, 7–CDs, 10–CDs and 14–CDs) were tested. In fluorescence spectra, it can be observed that the as-prepared CDs have different fluorescence emissions (Figure 2A). Under the same excitation wavelength (*λ*_ex_ = 360 nm), the maximum emission peak of 2–CDs is located at 492 nm, and the maximum emission peaks of 7–CDs, 10–CDs and 14–CDs are located at 434 nm. Although the maximum emission peak positions of 7–CDs, 10–CDs and 14–CDs are the same, the emission intensity and the shape of the emission peaks show noticeable differences. More notably, the intensity of the emission peak at 492 nm gradually increases with decreasing pH before the reaction. The pictures under sunlight and UV light more intuitively demonstrate the differences in the PL properties of the obtained CDs (Figure 2B). Under sunlight, it can be observed that the color of the CDs solution is obviously different. Under UV light, the PL color of CDs can be observed to gradually transition from green to blue with increase of the pH from 2 to 14, which is consistent with the fluorescence spectrum. The fluorescence quantum yields of the obtained CDs were also measured and compared. The quantum yields of 2–CDs, 7–CDs, 10–CDs and 14–CDs are 24.55%, 4.48%, 4.69% and 9.20%, respectively (Figure S2). The fluorescence quantum yields of 7–CDs and 10–CDs are significantly lower than those of 2–CDs and 14–CDs, which may be because CDs are more likely to generate surface defects that contribute to PL in strong acid or alkali environments. In summary, the PL properties of the CDs are affected by regulating the protonation degree of the carbon source m−PD. 

### 2.3. The PL Mechanism of CDs

**Figure 3:**
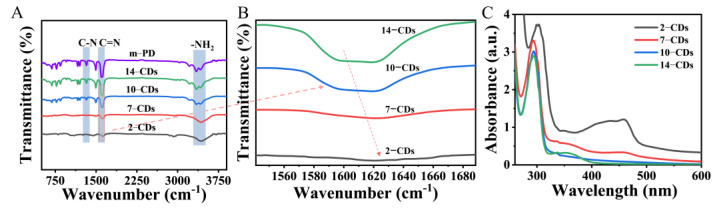
(**A**) FTIR spectrum of 2–CDs, 7–CDs, 10–CDs, 14–CDs and m−PD (the red arrow means to enlarge this part). (**B**) a magnified view of the C=N position in the FTIR spectrum of 2–CDs, 7–CDs, 10–CDs and 14–CDs (the red arrow represents the red-shifts). (**C**) UV absorption spectrum of 2–CDs, 7–CDs, 10–CDs and 14–CDs.

To reveal the reasons for the different PL properties, Fourier transform infrared (FTIR) spectra and UV–Vis absorption spectra of CDs were recorded. Compared with the m–PD molecule, the -NH_2_ vibrational absorption peaks at 3200 and 3340 cm^−1^ of 14–CDs, 10–CDs, 7–CDs and 2–CDs gradually weakened and disappeared in the FTIR spectra (Figure 3A), which indicates that the -NH_2_ of the CDs surface gradually decreases with the deepening of the protonation degree of the carbon source m–PD [21]. This can also be demonstrated by the weakening and disappearance of the C-N vibrational absorption peaks (1160 cm^−1^) with a decrease of the pH value. For clearer monitoring, we zoomed in on the FTIR spectrum at the C=N position. In Figure 3B, the position of C=N red shifts from 1606.9 cm^−1^ to 1625.3 cm^−1^ for 14–CDs to 2–CDs. The above phenomena indicate that with the deepening of the protonation degree of the source m−PD, the content of C=N double bonds increases [35]. The absorption characteristics of 2–CDs, 7–CDs, 10–CDs and 14–CDs were studied by UV-vis absorption spectra (Figure 3C). The absorption peaks at 292 nm were attributed to the π-π* transition of the benzene ring. The 2–CDs may be influenced by the abundant C=N functional groups on the surface, and the π-π* absorption peak representing the benzene ring has undergone a partial red shift to 298 nm [36]. The absorption peaks at 360 nm can be attributed to the n-π* transition of C-N bonds on the surface of CDs [37]. It can be observed that the n-π* transition peak at 360 nm gradually disappears with the deepening of the protonation degree of the carbon source m−PD. The absorption peak located at 458 nm representing the n-π* transition of the C=N bond gradually increased [25]. The UV–vis absorption spectra further confirmed the difference in the surface chemical bonds of CDs generated under different pH conditions. When the protonation degree of the carbon source m–PD gradually deepened, the C-N bonds on the surface of CDs gradually transformed into C=N bonds, which caused a series of changes in the UV–vis absorption and fluorescence spectra of CDs. 

**Figure 4:**
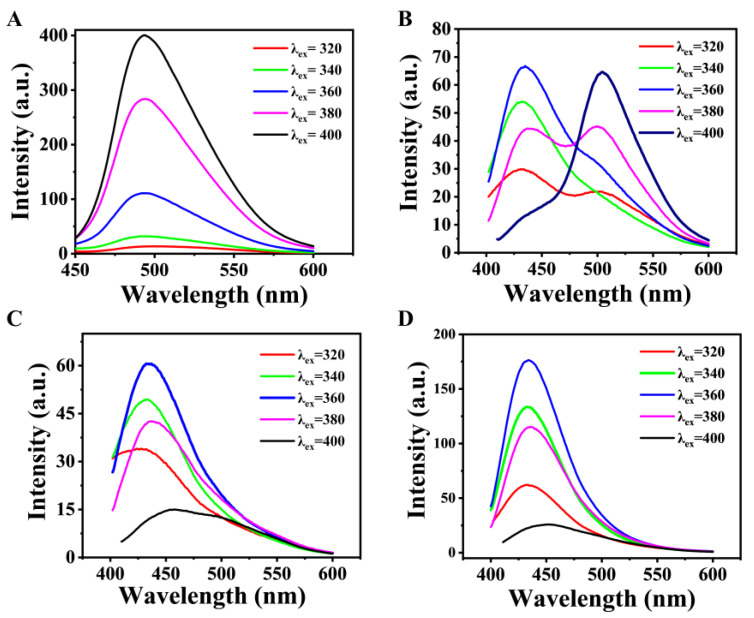
Fluorescence emission spectra of (**A**) 2–CDs, (**B**) 7–CDs, (**C**) 10–CDs and (**D**) 14–CDs at different excitation wavelengths.

Fluorescence-excitation dependence is an effective means to study the existence of different emission sites on the surfaces of CDs. Therefore, the fluorescence emission of 2–CDs, 7–CDs, 10–CDs and 14–CDs at different excitation wavelengths (320, 340, 360, 380 and 400 nm) were tested. Obviously, under different excitation wavelengths, the maximum emission peaks of 2–CDs and 14–CDs did not shift significantly, showing excitation independence (Figure 4A,D). This means that the fluorescence emission of 2–CDs and 14–CDs mainly comes from one emission site. In contrast, the maximum emission peaks of 7–CDs and 10–CDs shifted significantly at different excitation wavelengths (Figure 4B,C). In particular, 7–CDs showed emission peaks at 434 nm and 503 nm, which indicates the existence of different emission sites on the surface of 7–CDs. The relationship between the fluorescence emission wavelength and excitation wavelength of different CDs verifies that the surface chemical states of CDs synthesized at different pH values are different.

**Figure 5:**
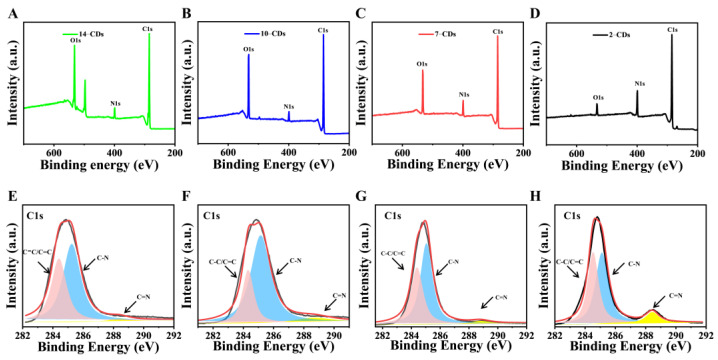
XPS survey spectrum of the (**A**) 14–CDs, (**B**) 10–CDs, (**C**) 7–CDs and (**D**) 2–CDs (the main peaks of C1s, N1s and O1s for the four CDs). High-resolution C 1 s of the (**E**) 14–CDs, (**F**) 10–CDs, (**G**) 7–CDs and (**H**) 2–CDs (pink, blue and yellow represent C−C/C=C, C−N and C=N, respectively).

The XPS measurement spectra of different CDs showed three main peaks located at 285.0, 399.0 and 532.0 eV, corresponding to C 1 s, N 1 s and O 1 s (Figure 5A–D). High-resolution XPS spectra of C 1 s (Figure 5E–H) confirmed the existence of C–C/C=C bonds (284.8 eV), C–N bonds (285.1 eV) and C=N bonds (288.4 eV). As the pH value decreased from 14 to 2, the protonation degree of carbon source m-PD deepened, which was accompanied by the surface functional groups of the as-prepared CDs gradually changing from mainly C–N bonds to C=N bonds. This is the reason for the different PL properties of CDs. The experimental data further indicate that the change of the surface chemical state of carbon atoms affects the PL phenomenon.

### 2.4. Application

#### 2.4.1. The Ratiometric Fluorescent Sensor

**Figure 6:**
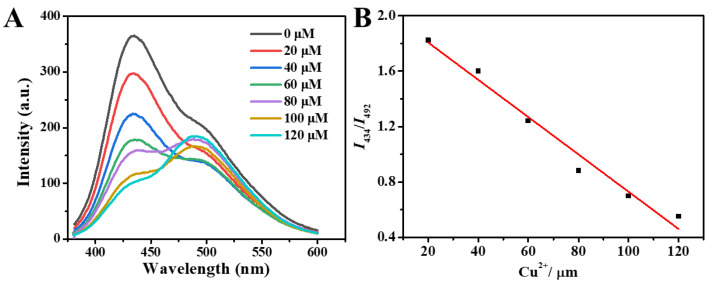
(**A**) Fluorescence emission spectra of 7–CDs at different Cu^2+^concentrations, (**B**) Cu^2+^ titration curve of the chemosensor for emission ratio at 434 and 492 nm (*I*_434_/*I*_492_). Indicated values are means of three measurements with the standard error of less than 3%.

Based on the surface-state-related PL properties of CDs, the application of CDs in different fields is realized. Due to dual fluorescence emission peaks of 7–CDs, which was used as a ratiometric fluorescent probe for Cu^2+^, it can be observed that the fluorescence intensity of 7–CDs at 434 nm gradually decreases with the increase of Cu^2+^ concentration, and the fluorescence intensity at 492 nm shows a trend of first decreasing and then increasing (Figure 6A). The fluorescent emission ratio of the 7–CDs at 434 and 492 nm (F_434_/F_492_) decreased linearly from 1.82 to 0.55 as the concentration of Cu^2+^ increased from 20 to 120 μM, with the following linear regression equation: *I*_434_/*I*_492_ = 2.076 − 0.013*c* (μM), *r*^2^ = 0.973 (Figure 6B). The decrease in fluorescence intensity at 434 nm caused by C−N can be attributed to the interaction of Cu^2+^ with amino groups on the surface of CDs, resulting in energy and electron transfer. The change of the fluorescence peak at 492 nm caused by C=N can be attributed to the coordination of Cu^2+^ with C=N and photoinduced electron transfer (PET). When the concentration of Cu^2+^ is low, the introduction of Cu^2+^ leads to the transfer of electrons in C=N, which inhibits its emission. However, when the concentration of Cu^2+^ is higher, the coordination of Cu^2+^ and C=N inhibits the PET phenomenon in C=N, which leads to the enhancement of the emission peak at 492 nm [38]. The response of 7–CDs to Cu^2+^ indicates its potential as a ratiometric fluorescent probe for Cu^2+^.

#### 2.4.2. Anti-Counterfeiting

**Figure 7:**
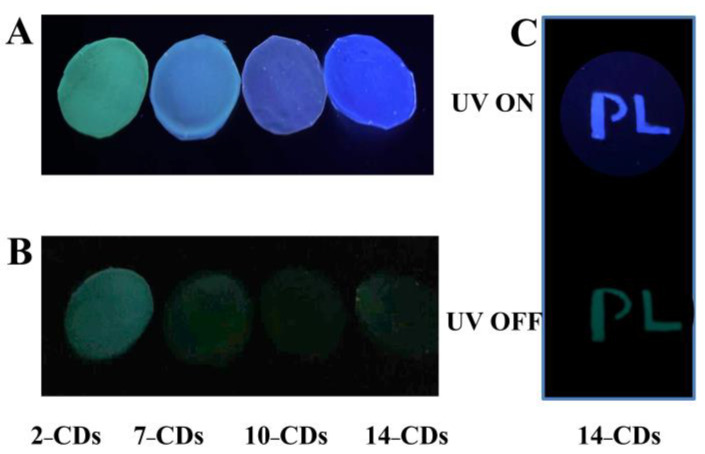
(**A**) Fluorescence and (**B**) Phosphorescence photos of 2–CDs−PVA, 7–CDs−PVA, 10–CDs-PVA and 14–CDs−PVA film, (**C**) 14–CDs−PVA film anti-counterfeiting application.

Since polyvinyl alcohol (PVA) can enhance the PL properties of CDs by isolating oxygen and external moisture, 2–CDs, 7–CDs, 10–CDs and 14–CDs as luminophores were dispersed in PVA to construct CDs–PVA PL composites films. Under UV excitation, it can be observed that all four composite films exhibit the dual emission properties of fluorescence and phosphorescence (Figure 7A,B). More importantly, the 14–CDs–PVA composite film emits blue fluorescence under UV excitation, while green phosphorescence can be emitted when UV excitation is turned off (Figure 7C). Based on this characteristic, 14–CDs-PVA composite is expected to be used in the field of double anti-counterfeiting.

## 3. Experimental Section

### 3.1. Materials

All chemicals used were analytical-reagent grade and were used as received, without further purification. m-PD, HCl, CuCl_2_ and NaOH were purchased from Beijing Chemical Reagent Company (Beijing, China). Polyvinyl alcohol (PVA, Mw = 1750 ± 50) was purchased from Macklin (Shanghai, China). Deionized water from the Millipore water purification system was used throughout the experiments.

### 3.2. Characterizations

High-resolution transmission electron microscopy (HRTEM) images were obtained by the JEOL JEM–ARM200F. X-ray diffraction measurements of powder were performed on a Bruck D8 Advance X-ray diffractometer equipped with graphite-monochromatized Cu/Kα radiation. The Raman spectrum was obtained by a LabRAM ARAMIS System with 785 nm laser radiation source (HORIBA Jobin Yvon, Paris, France). Transmission electron microscopy pictures were measured in EDX mode on a Tecnai G220 TEM (FEI Company, Hillsboro, OR, USA). X-ray photoelectron spectroscopy (XPS) measurements were performed by using an ESCALAB–MKII 250 photoelectron spectrometer (Thermo, Waltham, MA, USA). The luminescence spectra were performed on a F–7000 fluorescence spectrophotometer (Hitachi, Tokyo, Japan). The fluorescence quantum yield test was measured by FS–5 (Edinburgh, UK). The UV-vis absorption spectra was obtained on a Hitachi U-3900H spectrophotometer (Tokyo, Japan). Fourier transform infrared (FTIR) spectra data for the devices and powders were collected using a PerkinElmer Model 100 FTIR spectrometer (Waltham, MA, USA).

### 3.3. Preparation of CDs

0.20 g of m-PD was added to 50 mL of ethanol and sonicated for 10 min to dissolve it completely. The m-PD ethanol solution was added with hydrochloric acid/sodium hydroxide to adjust its pH to 2, 7, 10 or 14, placed in a reaction kettle with a Teflon lining and reacted at 180 °C for 15 h. Insoluble particles or impurities were removed to obtain a carbon dot solution. The obtained CDs were named 2–CDs, 7–CDs, 10–CDs and 14–CDs according to the pH value of the solution before the reaction.

### 3.4. Fabrication of CDs-PVA Film

The CDs (1 mL) obtained under different pH conditions were mixed with PVA (2 mL), stirred at room temperature for 1 h and the solvent was evaporated at 45 °C to obtain thin films.

### 3.5. Cu^2+^ Detection

In the Cu^2+^ detection experiment, 0 μM, 20 μM, 40 μM, 60 μM, 80 μM, 100 μM and 120 μM Cu^2+^ ethanol solutions were prepared. Next, 7–CDs (0.2 mg/mL) was added to the prepared Cu^2+^ ethanol solution, and mixed well. After standing for 1 h, the fluorescence emission spectrum was measured.

## 4. Conclusions

In summary, by adjusting the pH of the solution before the reaction (pH = 2, 7, 10 and 14), we changed the degree of protonation of the carbon source m–PD, which in turn affected the surface states of the CDs. The as-prepared CDs (2–CDs, 7–CDs, 10–CDs and 14–CDs) showed different luminescent properties. The mechanism study shows that with the increase of the protonation degree of m–PD, the C-N functional groups on the surface of CDs gradually decrease and the C=N functional groups gradually increase, which is the reason for the different PL properties of CDs obtained at different pH values. Furthermore, based on the differences in luminescence properties determined by different surface states, the applications of CDs in ion sensors and PL anti-counterfeiting were realized, respectively. The proposed method will provide an effective route to the controllable preparation of CDs with different PL properties.

## Data Availability

The data presented in this study are available on request from the corresponding author.

## Supplementary Materials

The following supporting information can be downloaded at: https://www.mdpi.com/article/10.3390/molecules27196517/s1, Figure S1. TEM images of (A) the 2–CDs, (B) 7–CDs, (C) 10–CDs and (D) 14–CDs. Inset: the photos of four CDs under ultraviolet light and their particle size distribution. Figure S2. Quantum yields of 2–CDs, 7–CDs, 10–CDs and 14–CDs.

## Sample Availability

Samples of the compounds 2–CDs, 7–CDs, 10–CDs, 14–CDs and m−PD are available from the authors.
