# Supplementary material for: Photoluminescence Mechanism of Carbon Dots: Triggering Multiple Color Emissions through Controlling the Degree of Protonation
Source: Molecules. 2022 Oct 2;27(19):6517. doi: 10.3390/molecules27196517 (PMC9571308; doi:10.3390/molecules27196517)
Supplement: Supplementary file 1 [file molecules-27-06517-s001.zip › molecules-1921594-supplementary.pdf]

Supporting Information

Photoluminescence Mechanism of Carbon Dots: Triggering Multiple Color Emissions through Controlling the Degree of Protonation

Hao Yi[1], Jing Liu[1], Jian Yao[1], Ruixing Wang[1], Wenying Shi[1]*, Chao Lu[1]

[1] State Key Laboratory of Chemical Resource Engineering, Beijing University of Chemical Technology, 15 Beisanhuan East Road, P.O. Box 98, 100029, Beijing (P. R. China).

* Correspondence: shiwy@mail.buct.edu.cn

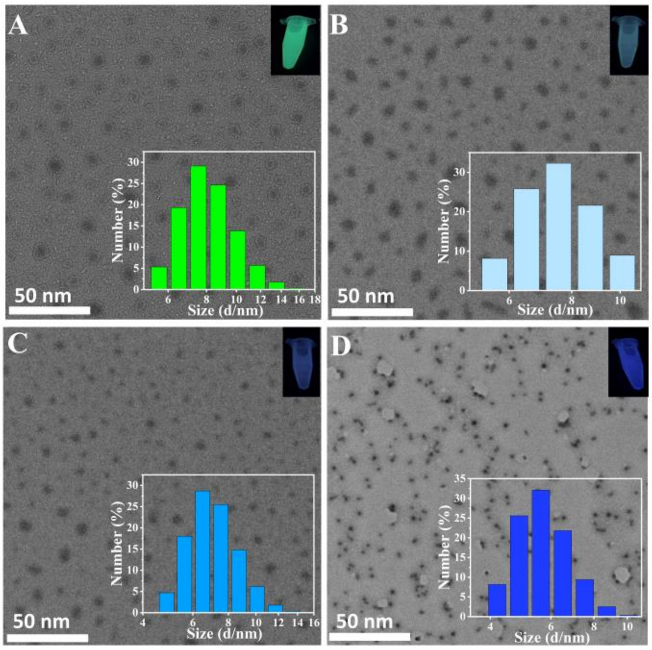

Figure S1. TEM images of (A) the 2-CDs, (B) 7-CDs, (C) 10-CDs and (D) 14-CDs. Inset: the photos of four CDs under ultraviolet and their particle size distribution.

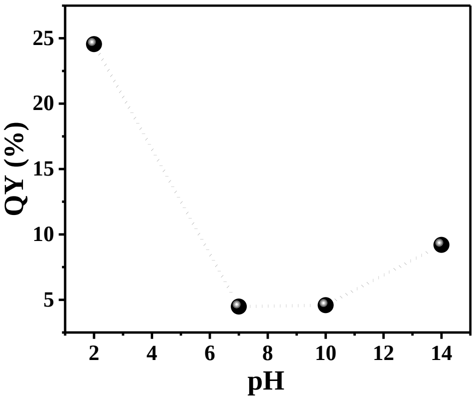

Figure S2. Quantum yields of 2-CDs, 7-CDs, 10-CDs and 14-CDs.
